# Novel *MBTPS1* variant associated with Kondo-Fu syndrome with expanded skeletal manifestations and a distinctive biochemical profile

**DOI:** 10.3389/fendo.2026.1951899

**Published:** 2026-10-27

**Authors:** Dorra Najjar, Aleš Maver, Imen Chelly, Asma Chikhaoui, Rim Boussetta, Sami Bouchoucha, Borut Peterlin, Houda Yacoub-Youssef

**Affiliations:** 1 Laboratory of Biomedical Genomics and Oncogenetics, LR16IPT05, Institut Pasteur de Tunis, Tunis El Manar University, Tunis, Tunisia; 2 Clinical Institute of Genomic Medicine, University Medical Centre Ljubljana, Ljubljana, Slovenia; 3 Department of Paediatrics, Habib Bougatfa Hospital, Bizerte, Tunisia; 4 Faculty of Medicine in Tunis, LR99ES10 Laboratory of Human Genetics, University of Tunis El Manar, Tunis, Tunisia; 5 Department of Pediatric Orthopedic Surgery, Bechir Hamza Children’s Hospital, Faculty of Medicine of Tunis, Tunis El Manar University, Tunis, Tunisia

**Keywords:** bone metabolism, Kondo-Fu syndrome, MBTPS1, novel splice site variant, osteopenia, phenotypic expansion, spondyloepiphyseal dysplasia, ultra-rare disease

## Abstract

**Introduction:**

Kondo-Fu syndrome is an ultra-rare genetic disorder caused by variants in the *MBTPS1* gene, which plays a key role in lysosomal function and skeletal homeostasis. Clinical presentation is characterized by spondyloepiphyseal dysplasia, low bone mineral density, and cataracts. This study aimed to expand the clinical spectrum by describing distinctive phenotypic features and highlighting novel genetic findings and biochemical alterations relevant to bone metabolism.

**Methods:**

We conducted extensive clinical, radiological (X-ray, 3D-CT scan, MRI), biochemical, and genetic investigations in a patient with Kondo-Fu syndrome in whom initial diagnostic evaluation was inconclusive. Whole-exome sequencing was performed on the proband, and the candidate variant was validated by Sanger sequencing in the family. We further evaluated its functional impact using real-time PCR, compared to two healthy donors.

**Results:**

Clinical investigations revealed the characteristic clinical features of Kondo-Fu syndrome, including osteopenia, spondyloepiphyseal dysplasia, cataracts, and intellectual disability. Furthermore, the patient presented previously unreported rare congenital anomalies, including os odontoideum, split atlas, and diaphragmatic hernia. Biochemical analysis revealed elevated alkaline phosphatase levels and decreased high-density lipoprotein and vitamin D levels, which could be associated with osteopenia and suggest altered bone and mineral metabolism. Genetic investigation revealed a novel homozygous variant, c.963 + 2T>A, in *MBTPS1*, leading to the diagnosis of Kondo-Fu syndrome, which was consistent with the patient’s clinical manifestations. Gene expression analysis demonstrated the under expression of *MBTPS1*, highlighting the deleterious effect of this variant.

**Conclusions:**

This study identifies a novel variant underlying Kondo-Fu syndrome and reveals previously unreported phenotypic features alongside exploratory biochemical findings that may provide preliminary insights into altered bone and mineral metabolism.

## 1 Introduction

Spondyloepiphyseal Dysplasia, Kondo-Fu Type (SEDKF) (OMIM: **#** 618392) is a rare genetic disorder, described for the first time by Kondo and Fu in 2018 (1). This syndrome is characterized by severe growth retardation, facial dysmorphism, bilateral cataracts, and skeletal anomalies, including spondyloepiphyseal dysplasia with associated kyphosis, pectus carinatum, valgus bowing, and reduced bone mineral density. Some patients present with bilateral inguinal hernias and intellectual disability. Elevated levels of blood lysosomal enzymes have also been observed in 40% of described patients (1–3). Currently, there are no therapies that target the cause of SEDKF. Patients can be managed with surgical and non-surgical treatments, such as surgical interventions to correct skeletal malformations and cataracts (1, 2).

This syndrome is often misdiagnosed and confused with mucolipidosis type II and III, Silver-Russell syndrome (SRS), or congenital spondyloepiphyseal syndrome. The differential diagnosis is based on genetic investigations.

Variants in the *MBTPS1* (membrane-bound transcription factor peptidase, site 1) gene (NM_003791.4) are associated with SEDKF with an autosomal recessive inheritance mode. This gene encodes Site-1 protease (S1P), a proprotein convertase subtilisin/kexin (PCSK8), a member of the serine protease family. This protein is ubiquitously expressed in the Golgi apparatus. It is the master regulator of various cellular functions via the regulation of three primordial processes of cellular metabolism: (i) regulation of intracellular cholesterol and fatty acid metabolism *via* cleavage of substrates at non-basic residues (4); (ii) endoplasmic reticulum (ER) stress response by cleaving and activating ATF6, which activates genes encoding ER chaperone proteins (5); and (iii) regulation of lysosomal enzyme transport and trafficking from the Golgi apparatus (6).

To date, only six cases of *MBTPS1* variants associated with Kondo-Fu syndrome have been reported (2). This ultra-rare syndrome is highly misdiagnosed as other types of skeletal dysplasia because of its rarity and clinical overlap, which makes it difficult to establish the correct diagnosis, and only genetic investigations can ensure the differential diagnosis. Four recent studies have associated *MBTPS1* gene variants with other syndromes, including cataract, alopecia, oral mucosa disorder, and psoriasis-like (CAOP) syndrome, as described by Chen et al. in two patients (8). Another study associated a *de novo* heterozygous variant with a complex phenotype characterized by episodic hyperCKemia, focal myoedema, and polycystic ovary syndrome in a single patient (9). Moreover, a study associated *MBTPS1* alterations with Silver-Russel syndrome (10), and a more recent study described it as an emerging novel disease (3).

Despite increasing reports of MBTPS1-related diseases, their clinical and genetic delineation remains incomplete. Especially for Kondo-Fu syndrome, where the clinical and genetic spectrum is still expanding (2, 11), highlighting the need for further characterization of its clinical and molecular features. Furthermore, lysosomal enzyme abnormalities have been largely reported in Kondo-Fu syndrome; however, comprehensive assessment of additional biochemical parameters related to low bone mineral density has been poorly explored. Therefore, a combination of in-depth clinical, biochemical, and genetic investigations is required to ensure accurate diagnosis. Furthermore, expanding the clinical and mutational spectrum through continued research is essential to advance our understanding of the disease and enhance diagnostic accuracy.

In this study, we report a patient harboring a novel *MBTPS1* variant, with detailed clinical investigation, where we report additional features, expanding the known spectrum and present further biochemical characterization. Hence, expanding the phenotypic and genetic spectrum of Kondo-Fu syndrome.

## 2 Materials and methods

### 2.1 Clinical data collection

This study was conducted following the Declaration of Helsinki principles and was approved by the Bio-medical Ethics Committee of the Pasteur Institute of Tunisia (Ethical Approval Reference 2021/10/E/V1). After obtaining written informed consent from the legal guardians of the minor patient, blood samples were collected in the pediatric orthopedic department of the Hospital Bechir Hamza. One proband was enrolled in the study and underwent routine general examinations for years. Clinical and genealogical data were collected under the supervision of the referring clinician. Radiography (X-rays, MRI, spine CT-scan) and biochemistry tests were performed for the patient suspected of spondylo-epimetaphyseal dysplasia.

### 2.2 DNA extraction

Blood samples were collected from the proband and his parents. Genomic DNA was extracted from peripheral blood mononuclear cells (PBMCs) using the FlexiGene DNA Kit, according to the manufacturer’s instructions.

### 2.3 Genetic investigation

Karyotype analysis was conducted for the patient as a routine diagnosis. Whole Exome Sequencing (WES) was conducted for the patient using the Twist Human-Core-Exome kit (manufactured by Twist Bioscience Corporation, South San Francisco, CA, USA). This was followed by sequencing on Illumina NovaSeq 6000 platforms.

The data obtained were subsequently analyzed using an internally developed pipeline based on the combined disease and phenotype gene target definition approach, as previously described (12–14). Basic analysis for the detection and annotation of Single Nucleotide Variant (SNV), canonical splice site variants, and small indels (insertion/deletion) was performed according to the GATK Best Practices workflow (15–17). Moreover, we employed additional data analysis methods to expand the spectrum of detected genetic variation and improve the identification of causative variants using the subsequent approaches: (a) identification of variations in noncanonical splice variants (18, 19). (b) Annotation and analysis of mitochondrial sequence, (c) copy number variants (CNV) analyses, (d) breakpoint analysis by detecting clusters of soft-clipped reads in aligned sequence, and (e) examination of the repeat expansions throughout the genome and the long runs of homozygosity (20, 21).

### 2.4 Gene selection

Our strategy was first based on creating a panel of genes based on the patient’s phenotype to identify relevant genes related to our genetic investigation. The list of genes was selected from different resources such as the human phenotype ontology (HPO) (13), OMIM (22), PubMed databases (23), PanelApp (24), and ClinGen (25) tools. This method ensured the selection of relevant genes related to our genetic investigation, thus enhancing the accuracy of our genetic analysis by focusing on a small number of variants. When no pathogenic variants were identified in this targeted panel, we expanded our investigation to encompass the full set of variants from the WES dataset, which ultimately allowed us to pinpoint the causative alteration.

### 2.5 Classification

The pathogenicity of the variants was evaluated according to the standards and guidelines provided by ACMG/AMP (American College of Medical Genetics and Genomics and the Association for Molecular Pathology) and the Association for Clinical Genomic Science (ACGS) criteria (26, 27).

### 2.6 Validation of the detected variant

The candidate variant was detected using WES, and the parental segregation was confirmed by the Sanger sequencing technique via ABI Prism 3130 sequencer (Applied Biosystems, Foster City, CA, U.S.A.). The primers used for the amplification were designed using Primer 3.0, based on the human *MBTPS1* gene sequence (forward: 5′- CCTTAGGTTCAGCTCCAAG − 3′ and reverse: 5′- CACTGAAGCTCACGTCATTA − 3′).

### 2.7 Functional validation of the variant effect

Total RNA was isolated from peripheral blood mononuclear cells of the patient, the patient’s mother, and two healthy age-matched donors using Trizol reagent (No: BCBL7327V, Sigma Aldrich, Darmstadt, Germany). The cDNA was obtained by reverse transcription using reverse transcriptase (Superscript II, #18064014, Invitrogen), according to the manufacturer’s instructions.

The cDNA was sequenced by Sanger sequencing to validate the effect of the variant on mRNA processing. The obtained sequences were aligned with the reference transcript to identify potential alterations in the exon–intron structure.We further tested the expression of the *MBTPS1* gene using SYBR Green-Based qPCR. Primers were selected from the PrimerBank database (https://pga.mgh.harvard.edu/primerbank/), based on the human *MBTPS1* gene sequence (forward: 5’-ACCTCGAAACAATCCATCCAGT -3’ and reverse: 5′- ACTTGAGGGAACGAAAGACTTTT -3’). The samples were tested in duplicate by qPCR using Light Cycler 480 SYBR Green I Master Mix (No: 04 707 516 001, Roche, Darmstadt, Germany), according to the manufacturer’s instructions. The qPCR was performed on the Light Cycler 480 System (Roche Diagnostics).

### 2.8 Statistics and reproducibility

Relative quantification Ct values were obtained from the threshold cycle numbers of a duplicate test. The normalization was performed using the ΔCt method relative to two housekeeping genes (*PPIA* and *RPLP0*), ensuring correction for sample-to-sample variation. The final results were presented as relative gene expression using the 2^-ΔΔCT^ cycle threshold method (28). We applied a fold-change threshold of 0.5 relative to the mean expression of housekeeping genes (corresponding to a 50% reduction) to define genes as underexpressed.

## 3 Results

### 3.1 General presentation of the patient

In this study, we report a case of a male patient (IV-8) who was referred to us for genetic counseling at the age of 15-year-old. He was born to healthy related parents from the Center-East of Tunisia who were first-degree cousins (**Figure 1**). The proband was referred to us for evaluation of dwarfism and skeletal deformities, leading us to suspect spondylo-epimetaphyseal dysplasia based on clinical and radiological findings.

**Figure 1:**
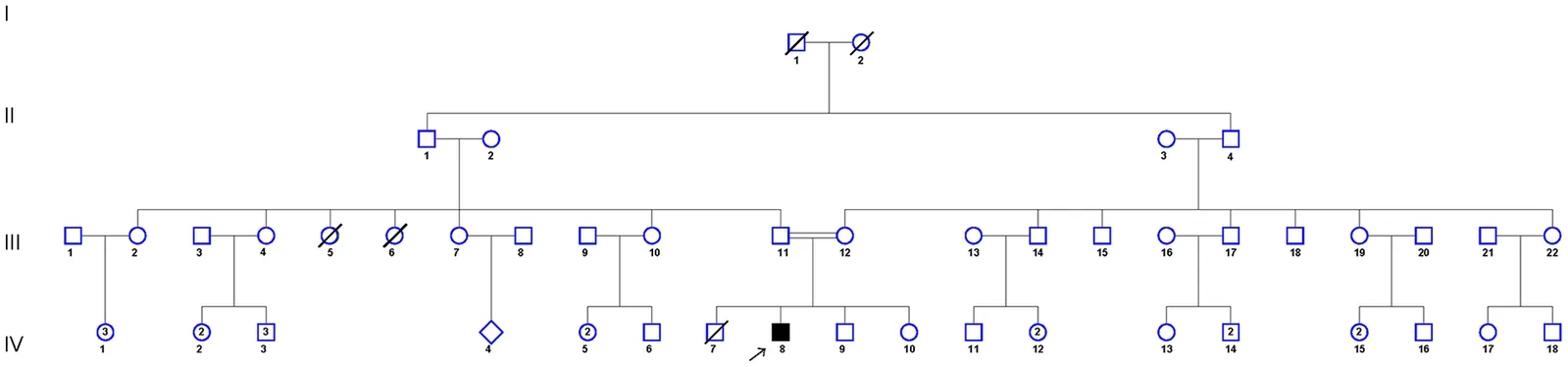
Pedigree of the affected family. Filled symbols represent affected individuals.

During the genetic inquiry, there was no family history of dwarfism or constitutional bone diseases; however, the family reported a single perinatal death due to complications during labor (IV-7).

### 3.2 Clinical investigations

Pregnancy and delivery were normal, with a low birth weight (2Kg200) and the height was not specified. He had a global developmental delay and intellectual disabilities, which were not investigated. At the age of three years old, the patient developed progressive skeletal deformities and postnatal growth retardation.

The patient was referred to us at the age of fifteen years; the clinical examination revealed dwarfism with a height of -11 standard deviations (SD), a weight of -5 SD, and an occipitofrontal circumference at -3.56 SD. He had facial dysmorphism with a prominent forehead, horizontal palpebral fissures, tubular nose, and large ears. The patient also had a small mouth and abnormal tooth implantation.

#### 3.2.1 Ocular anomalies

Ophthalmological examination revealed bilateral nystagmus and bilateral congenital cataracts that were surgically treated.

#### 3.2.2 Skeletal manifestations

The patient presented with a short neck, pectus carinatum, severe dorsal kyphoscoliosis, flessum of hips and knees, genu valgus deformity, short hands and feet with brachydactyly and joint hyperlaxity (**Figure 2**).

**Figure 2:**
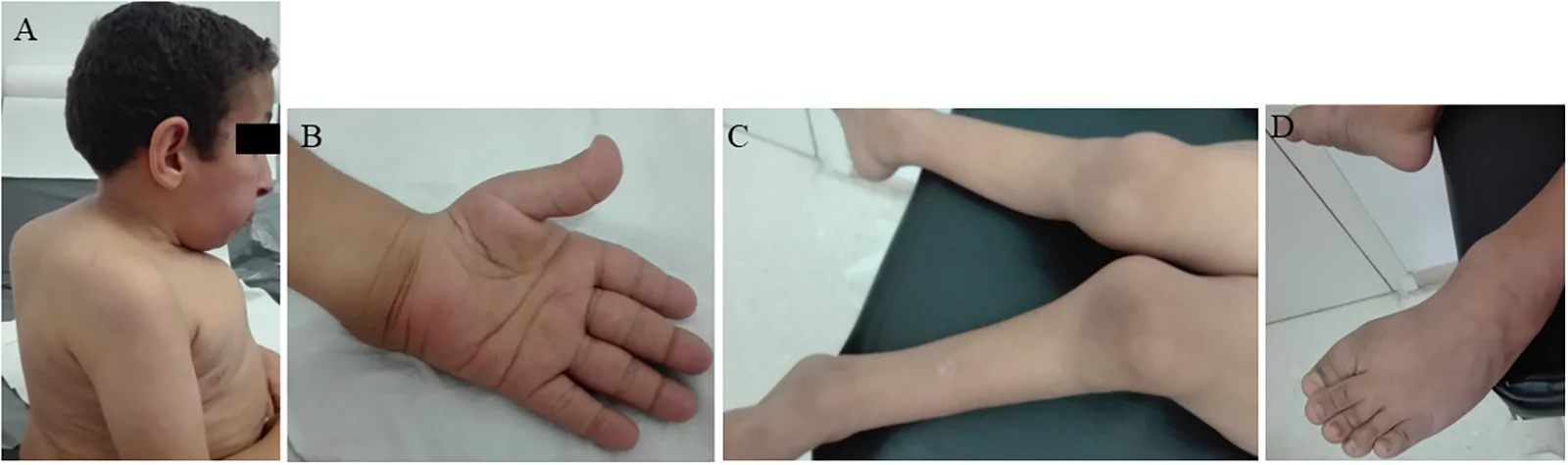
Photos of the clinical features of the patient. **(A)** prominent forehead, large ears, micrognathia, kyphosis, pectus carinatum; **(B)** small hand and brachydactyly; **(C)** Genu valgus; **(D)** Pes planovalgus.

Radiological investigations revealed osteopenia, spondylo-epimetaphyseal dysplasia with platyspondyly, subluxation of C1-C2, Split atlas, os odontoideum, causing atlantoaxial instability, congenital hemivertebrae involving the thoracic spine from T4 to T11, associated with spinal deformity, carpal dysplasia and brachymetacarpia (**Figure 3**).

**Figure 3:**
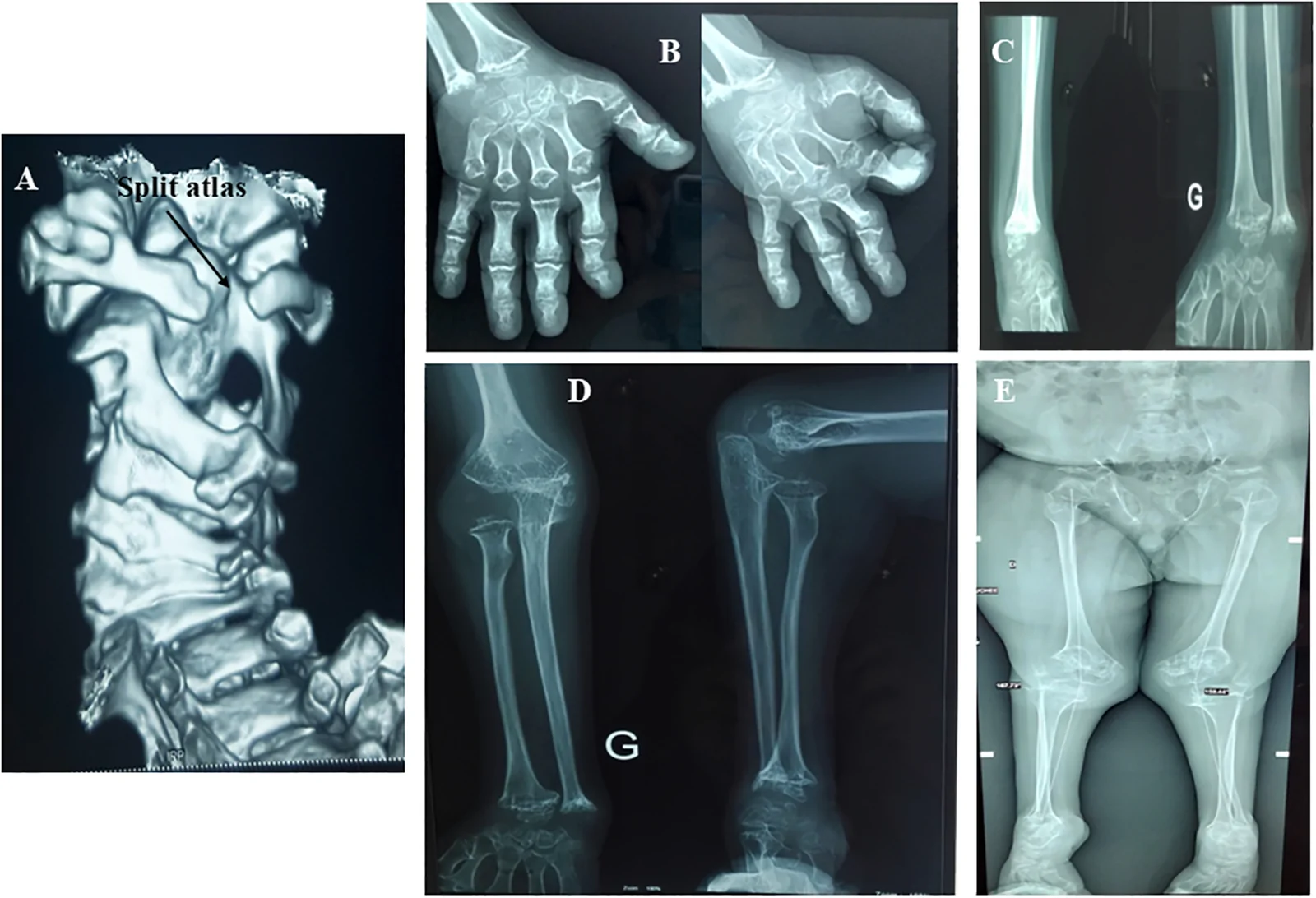
Radiological examination of the Kondo-Fu patient. **(A)** Split atlas; **(B)** Metacarpal dysplasia and brachydactyly; **(C)** Carpal dysplasia; **(D)** Radius bowing; **(E)** Hip dysplasia, bilateral shortening of the femoral neck, Irregular and dysplastic appearance of proximal and distal femoral, tibial epiphyses and metaphysis, and pes valgus.

#### 3.2.3 Neurological and behavioral abnormalities

The patient had a severe psychomotor delay. He acquired the ability to sit independently at the age of two and a half years, to stand up with support at the age of three years, and to walk with support at the age of four years. Due to progressive limb deformities, ambulation became increasingly challenging, ultimately resulting in the patient becoming wheelchair dependent. During follow-up, the patient underwent femoral osteotomy to correct genu valgus at 17 years of age, which subsequently allowed him to ambulate with a walker. In addition, the patient had a language delay acquisition and severe intellectual disability.

#### 3.2.4 Other clinical investigations

The patient presented with a left inguinal hernia, and magnetic resonance imaging revealed an anterior diaphragmatic defect, lateralized to the left, with intrathoracic herniation of the omentum, consistent with Larrey’s hernia. Echocardiography and abdominal ultrasonography findings were normal.

### 3.3 Treatments

The patient’s clinical management included a series of orthopedic interventions, such as combining a halo jacket with a Stagnara cast for kyphoscoliosis and cervical instability, and different surgical interventions to correct diaphragmatic and inguinal hernias, kyphoscoliosis, cervical bone instability, genu valgus, and cataracts.

### 3.4 Laboratory investigations

Laboratory tests revealed normal blood cell counts, creatinine, CRP, Plasma Protein Electrophoresis, blood urea nitrogen, aspartate aminotransferase (AST), alanine aminotransferase (ALT), bilirubin, and gamma-glutamyl transferase (GGT), TSH, FT4 levels, and Prothrombin Time. However, a high level of alkaline phosphatase (ALP), a low level of vitamin D (VD), and a low level of High-Density Lipoprotein (HDL) were detected. In addition, normal levels of urinary and blood creatinine, albumin, calcium, and electrolytes were detected. The urinary total mucopolysaccharides were within the normal range.

### 3.5 Genetic investigations

To determine the genetic etiology and make an accurate diagnosis, we performed Whole Exome Sequencing (WES) after obtaining a normal karyotype (46,XY). WES analysis revealed a novel homozygous variant at the canonical splice donor site (c.963 + 2T>A) of *MBTPS1*, which was further validated via Sanger sequencing (**Figure 4**) and confirmed in the parents for segregation with a heterozygous profile in both parents. We classified this variant as pathogenic, based on the ACMG criteria.

**Figure 4:**
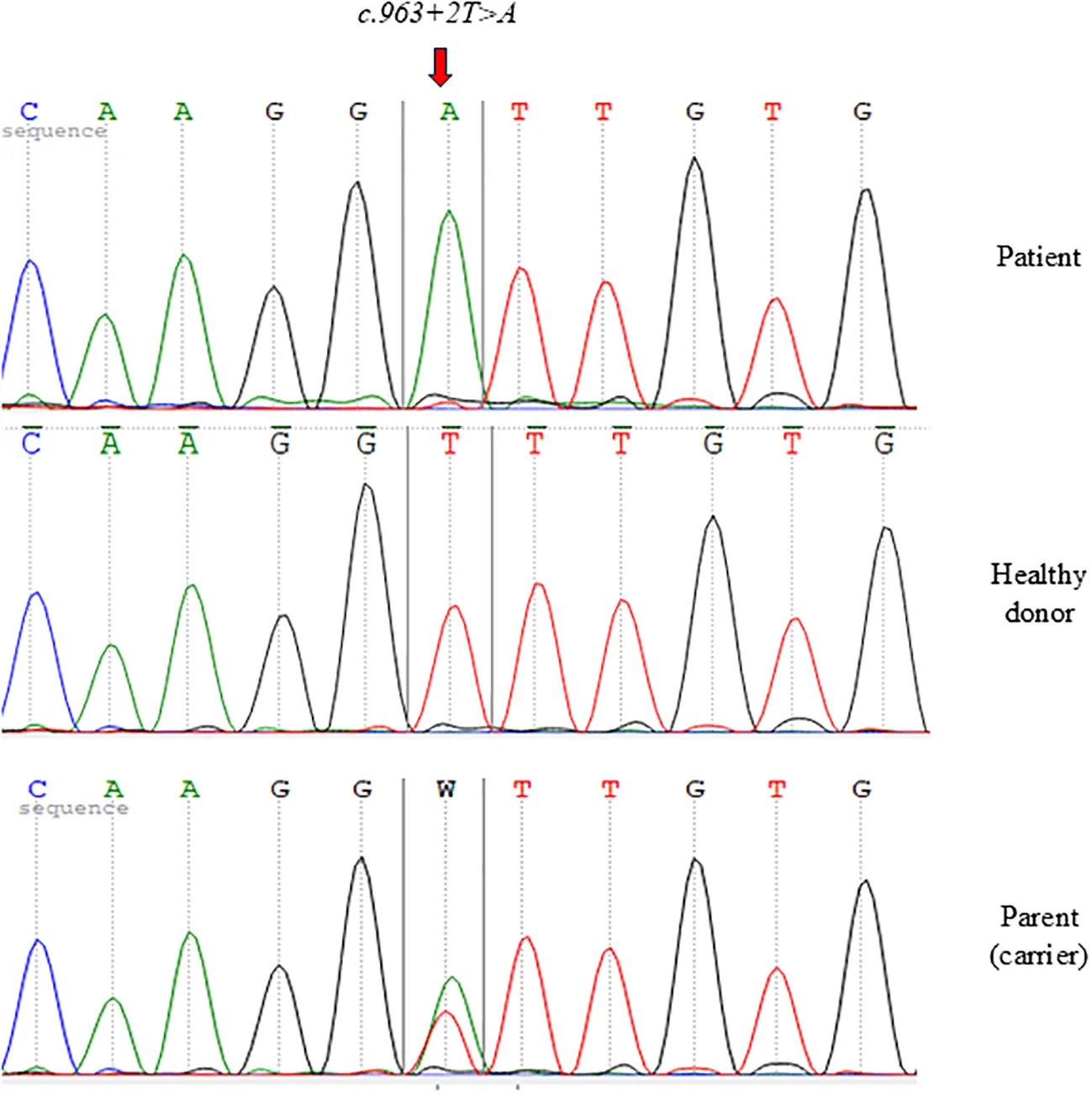
Electropherogram of the c.963 + 2T>A variant of the MBTPS1 gene. Electropherogram of the homozygous Kondo-Fu patient, the wild-type donor, and the heterozygous mother (carrier).

#### 3.5.1 In silico effect of the variant on the splicing site

This variant modifies the consensus donor splice site region in intron 7 of *MBTPS1*, changing the conserved GT to a GA motif. Prediction tools such as Human Splicing Finder (HSF), SpliceAI, Pangolin, and VARSEAK, etc, predicted a deleterious effect of the variant on the splicing process, supporting potential exon skipping and loss of the donor site (**Table 1**).

**Table 1:** Prediction of the pathogenicity of the splice site variant.

Prediction tools	Score	Classification
dbscSNV	0.9999	Pathogenic
EIGEN	1.0805	Pathogenic
EIGEN PC	0.9014	Pathogenic
FATHMM-MKL	0.9932	Pathogenic
SpliceAI	1.00	Donor loss
Pangolin	0.90	Splice loss
CLASS VARSEAK	Classe 5 +33.00%	Loss of function, Exon skipping
MaxEntScan	7.81>-0.38 (-104.87%)	Pathogenic
Maximum Entropy Model	-0.39	
Maximum Dependence Decomposition Model	3.99	
First-order Markov Model	-1.13	
Weight Matrix Model	-0.97	
Mutation Taster	1	- Disease-causing - Protein features (might be) affected - Splice site changes
Human Splicing Finder	86.47>59.33 (-31.39%)	Alteration of the WT donor site
CADD	31 (+2 points)	Moderate pathogenic

#### 3.5.2 Characterization of the splicing effect of the variant

To further investigate this prediction, we performed RT-PCR followed by Sanger sequencing of the resulting cDNA. The cDNA sequencing revealed an aberrant transcript containing retention of the intronic sequence adjacent to the affected exon. These results provide direct experimental evidence that the variant alters normal splicing.

#### 3.5.3 *MBTPS1* gene expression analysis

To functionally validate the effect of the splice variant on *MBTPS1* gene expression, mRNA was extracted from the patient’s PBMC, from the healthy mother, and from two healthy donors. Low gene expression was found in the healthy mother and especially in the patient (fold change = 0.39 and 0,051 respectively) compared to healthy donors (**Figure 5**).

**Figure 5:**
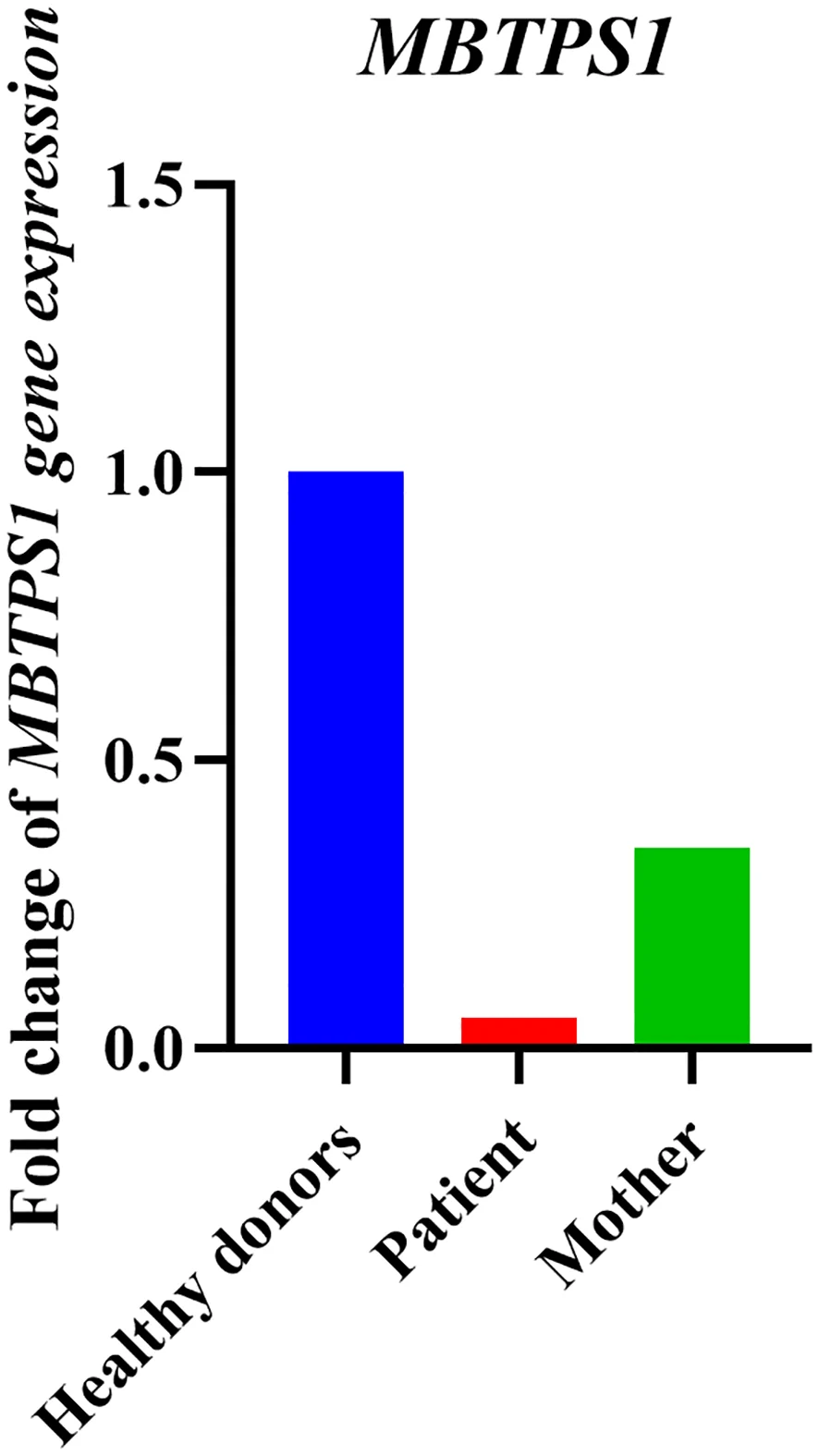
MBTPS1 gene expression in Kondo-Fu patient and his carrier mother. qPCR data are presented as the mean relative expression levels, normalized to the geometric mean of two housekeeping genes *RPLP0* and *PPIA*.

## 4 Discussion

### 4.1 Clinical findings

The diagnosis of skeletal dysplasia remains challenging because of its substantial clinical and genetic heterogeneity, along with phenotypic overlap with other disorders such as mucopolysaccharidoses, because of shared features such as short stature and skeletal deformities (29). This overlap frequently leads to initial misdiagnosis. In this study, we report a case that was initially suspected to have mucopolysaccharidosis type IV (Morquio syndrome) because of overlapping clinical features such as short stature and spondyloepiphyseal dysplasia. However, the urinary MPS level was within the normal range. Studies have shown that in atypical cases, urinary MPS analysis may not always be conclusive; therefore, lysosomal enzyme activity should be analyzed. In our case, it was not possible to carry out this analysis, and whole-exome sequencing was performed to establish a diagnosis (30, 31). Genetic investigation using WES identified a biallelic pathogenic variant in the *MBTPS1* gene in the patient, consistent with the clinical spectrum of spondyloepiphyseal dysplasia, type Kondo-Fu syndrome (SEDKF).

*MBTPS1* encodes the S1P protein, which is implicated in the trafficking of lysosomal enzymes from the Golgi apparatus to lysosomes, suggesting a potential role for MBTPS1 in lysosomal enzyme homeostasis. Beyond this function, S1P plays a role in type II collagen transport by controlling the formation of mega-vesicles, which are responsible for transporting large molecules such as collagen. A defect in collagen transport leads to an accumulation of collagen in chondrocytes, resulting in their apoptosis and an imbalance in the chondrogenesis process associated with the skeletal dysplasia observed in SEDKF patients (1, 2).

Kondo-Fu syndrome is characterized by growth retardation, spondyloepiphyseal dysplasia, kyphosis, chest deformity, delayed motor milestones, cataract, and facial dysmorphism (1). However, the clinical phenotype is still expanding with the discovery of diffused osteopenia, irregular appearance of metaphysis and epiphysis, ovoid lumbar vertebral bodies, small tubular epiphysis, and copper-beaten skulls as described observed by Carvalho et al. (11). As shown (Table 2, **Supplementary Table**), our patient shares several typical features with previously described cases, such as osteopenia, short neck, meta-epiphyseal dysplasia and pectus carinatum. Beyond its hallmark features, certain atypical skeletal anomalies not previously documented in the literature were observed as phenotypic particularities in our patient, such as cervical bodies abnormalities that were observed via a full-spine CT scan and MRI that demonstrated odontoid hypoplasia alongside an os odontoideum and Split atlas, which are rare cervical spinal anomalies causing atlantoaxial instability. These manifestations have been described in several disorders, such as Morquio syndrome, multiple epiphyseal dysplasias, Klippel-Feil syndrome, and Down syndrome (32, 33). In the present case, these phenotypes co-occurred with thoracic kyphoscoliosis, which has been described in only one patient in the literature, accompanied by a hemivertebra that has never been described in SEDKF patients, contributing to a broader and atypical clinical presentation. Moreover, we observed a widening of the perimedullary cerebrospinal fluid spaces associated with posterior vertebral scalloping, which were not present in the previous clinical case reports. These manifestations alongside platyspondyly led to spinal cord compression, which was treated via a combination of preoperative protocols employing a combined halo jacket with a Stagnara cast and surgical corrections of kyphoscoliosis and cervical instability. This protocol was established by our medical team and was the first to combine these two techniques in managing complex spinal deformities (34). Moreover, other rare skeletal manifestations were observed in our patients, such as brachydactyly, which was described in only two patients (2, 35). In addition, Carvalho et al. documented brachymetacarpia in one radiographic image, despite the absence of clinical evidence of brachydactyly (11). Genu valgum was also noted, resulting in walking difficulties that required assistance in one patient (35). Similarly, the severe valgus bowing observed in our patient led to an inability to walk, but after femoral osteotomy, the patient ambulates with a walker. Furthermore, radius bowing was observed only in one patient with humeral bowing (35), however, metacarpal dysplasia were never described in Kondo-Fu patients. The co-occurrence of these rare and unique manifestations suggests a potentially novel phenotypic continuum.

In addition to the rare skeletal manifestations, the patient presented with intellectual disability and speech delay, which are rarely associated with this syndrome (11, 35, 36). Notably, neurodevelopmental impairment in this case was severe and characterized by both intellectual disability and a severe delay in the acquisition of developmental skills, suggesting a more pronounced neurodevelopmental phenotype. This was observed in only one patient with delayed acquisition of sphincter control and speech (11). These findings expand the phenotypic spectrum and underscore the potential involvement of central nervous system dysfunction in this disorder. Moreover, we observed several phenotypes that have never been described as associated with this syndrome, which may contribute to the emerging phenotypic spectrum of the disorder. Dental crowding and joint hypermobility were described with the *MBTPS1* variants linked to the emergence of a novel syndrome described by Liaqat et al., 2024 (3), but were not associated with Kondo-Fu syndrome. In addition, diaphragmatic hernia has never been described in Kondo-Fu patients, nor have any studies implicated *MBTPS1* variants. In the absence of other variants that could explain these features in the WES data, we suggest that these clinical manifestations could contribute to the phenotypic spectrum of MBTPS1-related disorders. However, their occurrence in a single patient does not establish a causal association with MBTPS1 deficiency. Further reports of patients with Kondo–Fu syndrome will be needed to determine whether these features are consistently associated with this disease.

### 4.2 Molecular diagnosis

To date, 12 patients with MBTPS1-related disorders have been reported. Seven of these patients had Kondo-Fu syndrome (1, 2, 11, 35–38), two had CAOP syndrome, one had an emerging novel disease (3, 8), one had hyperCKemia and focal myoedema (9), and one had SRS (10). The number of Kondo–Fu patients may vary depending on the classification of cases with overlapping *MBTPS1*-related phenotypes. In particular, Lucas-Castro et al. (38) considered the patient reported by Meyer et al. (2020) as a Kondo-Fu case, whereas Meyer et al. and subsequent reports by Alotaibi et al. (2022) and Yuan et al. (2022) did not classify this patient as diagnosed with Kondo–Fu syndrome. In the present review, we therefore classified this patient separately as an SRS phenotype. This study presents a novel homozygous donor splice site variant c.963 + 2T>A in the *MBTPS1* gene. This variant is located within the peptidase S8 catalytic domain of the protein, potentially leading to functional impairment. In the literature, twelve variants in the *MBTPS1* gene have been associated with Kondo-Fu syndrome, and most of these variants are located in this protein domain (2). Herein, we present the second case with a canonical splice site variant associated with Kondo-Fu syndrome, in addition to the previous one (38). Moreover, two cases of cryptic splice site variants have been reported, leading to exon skipping and nonsense-mediated mRNA decay (NMD) (1, 36). Similarly, based on in- silico prediction tools, this variant was predicted to cause exon skipping. However, cDNA Sanger sequencing of the patient revealed an intron retention, demonstrating an abnormal splicing event associated with the variant. In addition, the markedly reduced expression of the *MBTPS1* gene (~5% of control level) in the patient and the reduced expression at approximately 39% compared to healthy controls in the heterozygous mother, suggests that this splicing variant may affect transcript stability, potentially through nonsense-mediated mRNA decay (NMD), or results in protein truncation. A similar effect was reported by Kondo et al., where the splice site variant resulted in ~1% expression of this gene in the patient and 40% in the healthy mother (1). This supports that the loss-of-function mechanism is associated with Kondo-Fu syndrome (1, 11); however, gain-of-function variants in *MBTPS1* are associated with episodic hyperCKemia and focal myoedema (9). Our findings are consistent with this report and provide further evidence supporting that loss-of-function variants in *MBTPS1* are linked to the pathogenesis of SEDKF, while expanding the functional spectrum of splicing alterations linked to this disease.

Although no specific treatment is currently available for this syndrome, some studies have demonstrated the potential efficacy of antisense oligonucleotide therapy using morpholino oligonucleotide as a modulator of splice site variants in the restoration of endoplasmic reticulum activity and collagen I secretion in fibroblasts obtained from Kondo-Fu patients with splice site variants (1, 36). This could serve as a potential therapeutic strategy for patients with splice site variants, such as the patient described in this report.

### 4.3 Biochemical particularities

Normal blood test results were observed for the main bone metabolism indicators, including serum calcium, phosphorus, ALT, AST, GGT, electrolytes, and parathyroid hormone, all of which were normal, consistent with other studies. However, we found a high level of alkaline phosphatase (ALP) in the blood; this result was similar to that of only one patient described by Chen et al. (37), who had alkaline phosphatase slightly above the normal range; nevertheless, most Kondo-Fu patients present normal values of ALP (11, 35, 36). Indeed, alkaline phosphatases are enzymes found in many body tissues, 95% of which are secreted by the bone and liver. Altered blood levels generally reflect bone or liver pathologies (39). In our patient, normal levels of liver enzymes are observed, and together with the presence of osteopenia, we suggest that the elevated ALP predominantly originates from the bone, indicating underlying bone pathology. In fact, alkaline phosphatase is an important hydrolase enzyme related to osteoblastic activity, and alterations in its secretion are negatively correlated with low bone mineral density, leading to osteoporosis (40).

Furthermore, the patient exhibited low vitamin D (VD) levels, consistent with findings reported in another SEDKF patient (36). In a previous study, Chen et al. supplemented their patient with vitamin D and vitamin A (37); nevertheless, other Kondo-Fu patients presented normal values of VD (11, 35). Vitamin D is essential for bone homeostasis and is involved in bone growth and bone remodeling by osteoblasts and osteoclasts, and its deficiency accelerates bone turnover, promotes bone loss, and causes osteoporotic fractures (41).

In addition, we found a low level of HDL in the blood test of our patient; only two patients in the literature described a low level of HDL (1, 36). A negative correlation between HDL and bone mineral density (BMD) has been described in the literature, contributing to the increased risk of osteoporosis and suggesting a role for HDL metabolism in bone homeostasis (42, 43). Experimental studies have indicated that reduced or dysfunctional HDL contributes to bone loss by promoting inflammation and impairing osteoblast differentiation (44).

Although these observations remain preliminary, these metabolic alterations in HDL, ALP, and vitamin D observed in our patient may lead to increased bone turnover and bone loss by reducing bone mineral density and may have contributed to the observed osteopenia in addition to the genetic defect. In fact, S1P plays an important role in bone formation by cleaving the FAM20C propeptide, enabling its activation and secretion into the Golgi apparatus. This protein kinase promotes osteoblast differentiation and biomineralization (7). Therefore, the alteration of the S1P-FAM20C pathway could play a potential mechanistic role in the low bone mineral density observed in Kondo-Fu patients. However, these biochemical alterations could be influenced by environmental factors such as sunlight exposure, nutrition, and mobility. Accordingly, for these patients, close monitoring of BMD and early intervention via correcting these metabolic alterations may be necessary to reduce future complications such as osteoporotic fractures, thereby enhancing the patient’s quality of life. However, further multicenter studies are needed to validate whether these biochemical findings are associated with Kondo-Fu syndrome. Overall, our study reported the first patient in North Africa and may expand the genetic and phenotypic spectrum of Kondo-Fu syndrome by identifying a novel genetic variant, describing clinical feature particularities that have not been previously reported, and showing biochemical alterations that could be associated with osteopenia observed in our patient. These findings further highlight the importance of combining clinical, biochemical, and genetic studies to better understand this ultra-rare disorder.

However, this study is based on one patient, and the eighth patient in the world according to our classification criteria, reflecting both the ultra-rare nature of Kondo-Fu syndrome and the potential underdiagnosis of this disease. Clinical and biochemical observations are limited to our patient, while some features have been described in other patients, which restricts the generalizability and association of our findings to this disorder. In addition, biochemical findings could be related to environmental factors and may influence bone metabolism, which may influence some clinical features. Although the reduced expression of the *MBTPS1* gene supports the deleterious effect of the variant, a functional study is needed to determine the splicing defect. Further studies in larger cohorts, supported by functional assays, will be necessary to determine the reproducibility of our observations and to elucidate the biological role of MBTPS1, in addition to the biochemical alterations in bone metabolism and homeostasis disturbance related to this disorder hemic.

## Conclusion

The present study represents the first North African Patient with Kondo-Fu syndrome. It contributes to the current understanding of the clinical spectrum of this disease by describing previously unreported features observed in our patient, hemic and underscores the relevance of genetic investigations to reach an accurate diagnosis. Furthermore, this study describes a novel homozygous *MBTPS1* pathogenic variant, further broadening the mutational spectrum. We further explored several biochemical parameters and identified potential factors that may provide preliminary clues regarding the mechanisms underlying the osteopenic phenotype observed in some patients, warranting further investigation in additional patients. Collectively, our findings emphasize the value of systematic characterization and a multidisciplinary approach for establishing precise diagnoses of ultra-rare bone diseases and in guiding appropriate patient care.

## Data Availability

The datasets presented in this article are not readily available because they contain individual-level genomic data from a single patient, and their sharing is restricted under the informed consent obtained and the ethical framework of Institut Pasteur de Tunis (approval 2021/10/E/V1). De-identified data may be made available to qualified researchers upon reasonable request to the authors, subject to institutional and ethical approval. The variant identified in this study has been submitted to the ClinVar database (NCBI) under accession number SCV007984513.
