# Supplementary material for: Novel MBTPS1 variant associated with Kondo-Fu syndrome with expanded skeletal manifestations and a distinctive biochemical profile
Source: Front Endocrinol (Lausanne). 2026 Oct 27;17:1951899. doi: 10.3389/fendo.2026.1951899 (PMC13747878; doi:10.3389/fendo.2026.1951899)
Supplement: Supplementary file 1 [file Table1.docx]

Supplementary Material

Table 1: Comparative overview of the clinical features in the Tunisian patient and previously reported cases of Kondo-Fu syndrome

| **Patient code**  **Clinical features** | **This study** | **(1)** | **(11)** | **(35)** | **(36)** | **(37)** | **(2)** | **(38)** | **proportion** |
| --- | --- | --- | --- | --- | --- | --- | --- | --- | --- |
| Number of patients | 1 | 1 | 1 | 1 | 1 | 1 | 1 | 1 | 8 |
| Consanguinity | + | - | + | + | - | - | - | - | 3/8 |
| Sexe | Male | Female | Female | Female | Male | Male | Female | Male | 4M / 4F |
| Age at initial evaluation (years) | 15 | 19 | 5 | 10 | 12 | 6 | 4 | 20 | Mean age at first diagnosis: 11 |
| **GROWTH** | | | | | | | | | |
| - Short stature | 83 cm (-11 DS) (severe nanism) | 162 cm (-2.72SD) | 79 cm  (-6.9 SD) | 102 cm (-2.9 SD) | 137.4 cm (-2.5SD) | 99 cm (-3.96SD) | + | 133.5 (-10.05 SD) | 8/8 |
| - Low weight | 22 Kg (-5 SD) | 62 kg  (-0.96 SD) | 9kg  (-8.8 SD) | 16 kg (-3 SD) | 40 kg  (-0.68 SD) | 12.80 kg (-2.84SD) | + | 26.2 kg (-4.21 SD) | 8/8 |
| - Full-term gestational age | Full term | Full term | 34 weeks | Full term | Full term | Full term | 36 weeks | 28 weeks | 5/7 |
| **HEAD & NECK** | | | | | | | | | |
| Face | | | | | | | | | |
| - Prominent forehead  - Microcephaly OFC | +  50cm (-3.56SD) | -  - (−0.67 SD) | +  47.5 cm (<3 SD) | +  48cm (<3 SD) | -  NI | +  NI | +  - | Broad forehead 52.8 cm (−2.38 SD) with relative macrocephaly. | 5/8  4/6 |
| Eyes | | | | | | | |  |  |
| - Congenital bilateral cataracts | + | + | + | - | + | + | + | + (unilateral) | 7/8 |
| *Mouth* | | | | | | | |  |  |
| Mouth size | Small | Normal | Wide | NI | NI | NI | NI | NI |  |
| Micro/retrognathia | + | - | + | + | + | + | + | + | 7/8 |
| Abnormal tooth placements | **+** | - | - | NI | NI | NI | - | NI | 1/4 |
| Ears | | | | | | | |  |  |
| - Prominent ears - Posteriorly rotated ears | +  **-** | +  - | +  - | +  - | +  - | -  + | +  - | +  - | 6/7  1/7 |
| *Neck* | | | | | | | |  |  |
| Short neck | + | - | + | + | - | - | + slightly | NI | 4/7 |
| **THORACIC** | | | | | | | |  |  |
| - Pectus carinatum | + | + | + | + | + | - | + | + | 7/8 |
| -Widely spaced nipples | **+** | NI | + | NI | NI | NI | NI | NI | 2/2 |
| -Diaphragmatic hernia | **+** | - | - | - | - | - | - | - | 1/8 |
| - Excessive bone calcification | **+** | NI | NI | NI | NI | NI | NI | NI | 1/1 |
| **GENITOURINARY** | | | | | | | |  |  |
| - Inguinal hernias | + (unilateral) | + (bilateral) | - | - | + (bilateral) | - | + (bilateral) | + | 5/8 |
| **SKELETAL** | | | | | | | |  |  |
| - Low bone mineral density | + (osteopenia) | + | + | + | + | + | NI | + | 7/7 |
| Spine | | | | | | | |  |  |
| - Kyphosis/scoliosis | + Severe dorsal kyphoscoliosis of 68° Cobb | kyphosis | kyphosis | kyphosis | Scoliosis | kyphoscoliosis | - | S/coliosis | 2/8 kypho-scoliosis  2/8scoliosis  3/8 kyphosis |
| - Cervical bone instability | + C1-C2 | NI | NI | NI | NI | NI | NI | NI | 1/1 |
| - Split atlas | + | NI | NI | NI | NI | NI | NI | NI | 1/1 |
| - Os odontoideum | + | NI | NI | NI | NI | -NI | NI | NI | 1/1 |
| - Vertebral bone dysplasia | + | NI | + | NI | + | + | + | + | 6/8 |
| - Platyspondyly | + | NI | + cervical | NI | + | NI | NI | + | 4/4 |
| - Hemivertebrae | + (D4-D11) | NI | NI | NI | - | NI | NI | NI | 1/2 |
| - Abnormality of the perimedullary cerebrospinal fluid spaces | Widening of the perimedullary cerebrospinal fluid spaces, causing posterior vertebral scalloping | NI | NI | NI | NI | NI | NI | NI | 1/1 |
| Limbs | | | | | | | |  |  |
| - Bilateral shortening of femoral necks | + | + | + | - | - | - | + | + | 5/8 |
| - Irregular and dysplastic appearance of femoral epiphyses | + | + | + | + | + | - | + | + | 7/8 |
| - Hip dysplasia | **+** | NI | NI | + | NI | NI | - | + | 3/4 |
| - Irregular and dysplastic appearance of proximal and distal tibial epiphyses and metaphysis | **+** | + | + | + | - | - | - | + | 4/8 |
| - Gracile fibulae | - | + | NI | NI | NI | NI | - | - | 1/3 |
| - Genu valgus | **+** | - | - | + | - | - | - | + | 3/8 |
| - Delayed ossification of epiphyses / carpal | NI | + | - | + | - | + carpal | - | + epiphyses and patella | 3/8 |
| - Shortening of tubular bones | + | + | + | + | - | - | + | + | 6/8 |
| -Radius bowing | **+** | NI | - | + | NI | NI | NI | + | 3/4 |
| - Foot deformities | **+** Pes planovalgus | - | - | + Pes Cavus | - | + Pes valgus | - / Pied bot | + Pes planus | 5/8 |
| Hands | | | | | | | |  |  |
| - Brachydactyly  - Brachymetacarpia  - Carpal and Metacarpal dysplasia | +  +  + | -  -  NI | -  +  NI | +  -  NI | -  -  NI | -  -  NI | +  -  NI | -  NI  NI | 3/8  2/7  2/7 |
| *Joints* | | | | | | | |  |  |
| Ligamentous hyperlaxity | **+** | NI | NI | NI | Hyper extended fingers | NI | NI | + | 3/8 |
| **NEUROLOGIC** | | | | | | | |  |  |
| Central Nervous System | | | | | | | |  |  |
| - Delayed gross motor milestones  - Speech delay  - Delayed acquisition of sphincter control  - Intellectual disability | +  **+**  **+** (at the age of 11 years old)  Severe | +  -  -  - | +  +  +  + | +  +  Mild | -  -  NI  Mild | -  -  -  - | -  -  NI  - | +  -  NI  - | 5/8  3/8  3/7  4/8 |
| **LABORATORY ABNORMALITIES** | | | | | | | |  |  |
| - Elevated lysosomal enzymes | NI | + | + | + | NI | NI | NI | -/+ | 4/4 |
| - Elevated alkaline phosphatase | **+** | - | - | - | - | + | NI | NI | 2/6 |
| - Vitamin D insufficiency | + | - | - | - | + | NI | NI | NI | 2/5 |
| - Low level of High-Density Lipoprotein (HDL) | + | + | NI | NI | + | NI | NI | NI | 3/3 |
| - Elevated mucopolysaccharides | - | NI | NI | NI | NI | NI | NI | NI | - |

+/-: Presence / absence; M/F: Male / Female; NI: non-identified
